# Mobile phone-based surveillance for animal disease in rural communities: implications for detection of zoonoses spillover

**DOI:** 10.1098/rstb.2019.0020

**Published:** 2019-08-12

**Authors:** Samuel M. Thumbi, M. Kariuki Njenga, Elkanah Otiang, Linus Otieno, Peninah Munyua, Sarah Eichler, Marc-Alain Widdowson, Terry F. McElwain, Guy H. Palmer

**Affiliations:** 1 Paul G Allen School for Global Animal Health, Washington State University, Pullman, WA 99164-7090, USA; 2 Center for Global Health Research, Kenya Medical Research Institute, PO Box 1578-4100, Kisumu, Kenya; 3 Washington State University—Global Health Program, Washington State University, PO Box 72938-00200, Nairobi, Kenya; 4 Division of Global Health Protection, Center for Global Health, Centers for Disease Control and Prevention, PO Box 606-00621, Nairobi, Kenya; 5 Division of Global Health Protection, Center for Global Health, Centers for Disease Control and Prevention, Atlanta, GA 30333, USA

**Keywords:** zoonoses, One Health, community-based surveillance, emerging infections

## Abstract

Improving the speed of outbreak detection and reporting at the community level are critical in managing the threat of emerging infectious diseases, many of which are zoonotic. The widespread use of mobile phones, including in rural areas, constitutes a potentially effective tool for real-time surveillance of infectious diseases. Using longitudinal data from a disease surveillance system implemented in 1500 households in rural Kenya, we test the effectiveness of mobile phone animal syndromic surveillance by comparing it with routine household animal health surveys, determine the individual and household correlates of its use and examine the broader implications for surveillance of zoonotic diseases. A total of 20 340 animal and death events were reported from the community through the two surveillance systems, half of which were confirmed as valid disease events. The probability of an event being valid was 2.1 times greater for the phone-based system, compared with the household visits. Illness events were 15 times (95% CI 12.8, 17.1) more likely to be reported through the phone system compared to routine household visits, but not death events (OR 0.1 (95% CI 0.09, 0.11)). Disease syndromes with severe presentations were more likely to be reported through the phone system. While controlling for herd and flock sizes owned, phone ownership was not a determinant of using the phone-based surveillance system, but the lack of a formal education, and having additional sources of income besides farming were associated with decreased likelihood of reporting through the phone system. Our study suggests that a phone-based surveillance system will be effective at detecting outbreaks of diseases such as Rift Valley fever that present with severe clinical signs in animal populations, but in the absence of additional reporting incentives, it may miss early outbreaks of diseases such as avian influenza that present primarily with mortality.

This article is part of the theme issue ‘Dynamic and integrative approaches to understanding pathogen spillover’.

## 1. Introduction

Local, regional or global spread of emerging infectious diseases (EID) can have severe impact on human and animal health, cause large economic losses and threaten health security [1,2]. Faced with these global health threats, research has focused on understanding sources of EID, patterns and drivers for their emergence and spread, prediction and detection of EID outbreaks and effective strategies for their prevention and control.

Nearly two-thirds of EIDs are of zoonotic origin [3,4]. The frequency of EID events has increased significantly over time driven by socio-economic, environmental and ecological factors such as rural-to-urban migrations resulting in high population density peri-urban settlements, intensive animal farming and trade, ease in travel, human-induced environmental changes such as widespread forest clearance and climate change, political instability and breakdown of public health measures [1,4,5].

Although determining the next new pathogen that will emerge, or the exact location where the next EID event will occur remains difficult, actionable information includes that an EID is likely to be of animal origin, occur in areas with high human–animal interactions, weak health systems and where the drivers of EID emergence converge [6–8]. Hotspots for emerging zoonotic diseases are predicted to be concentrated in the lower-latitude developing countries, regions that have relatively weaker surveillance systems in the human and animal health sectors and are most likely to suffer the largest impact from EID events [4,9]. For example, a review of all outbreaks reported to the World Health Organization between 1996 and 2009 reported that 53% of all EID outbreaks occurred in Africa [6]. Prediction, early detection and rapid response with effective tools such as vaccines are viewed to be our best tools to prevent and control EID [2,7,10,11].

Prediction and early detection for EID and their spread are dependent on obtaining ‘natural history’ disease data to understand transmission parameters such as infectiousness, latency, incubation and infectious periods, and surveillance data that describe spatial and temporal patterns of the diseases [12]. The complexity and difficulty in obtaining these data and the solutions to strengthening disease surveillance in developing countries are discussed in detail elsewhere, including focus on endemic diseases to increase capacity to detect and contain EID [13–16]. Taken together, there is a clear basis for surveillance of these diseases, focused on low- and middle-income countries with low biosecurity and high human–livestock–wildlife interactions, that promptly captures disease events happening at the community level and that relays these data in real or near real time for prompt public health response.

Mobile phones, which are now ubiquitous globally including in rural Africa, have provided opportunities to improve medical and public health practice including surveillance data collection, communication and delivery of preventive or restorative care [17–22]. Apart from using human healthcare and veterinary workers to collect and submit surveillance data, there has been interest in crowdsourcing data to rapidly detect outbreaks using mobile phone or Internet-based surveillance systems, including use in the 2014–2015 Ebola outbreak in west Africa [23–25].

These phone-based surveillance systems capture higher numbers of disease cases compared to traditional health facility or veterinary office-based surveillance systems. But significant concerns on how to verify or corroborate such data, and the risk of reporting bias based on access to phones or the Internet or influenced by social, economic and behavioural variations within the population have been raised.

Here, we evaluate the use of mobile phone-based syndromic surveillance for diseases in a rural community in Kenya, comparing it with active surveillance through routine household visits, and determine the individual and household correlates of their use. Using examples of select zoonotic diseases, we examine the broader implications of such a system for the effective surveillance of zoonotic infectious diseases.

## 2. Methods

### (a) Study population

Data used in this study came from 1500 households comprising 6400 persons participating in a linked human–animal syndromic surveillance study within a Health and Demographic Surveillance System in western Kenya (see details in [26]). Briefly, the linked human–animal surveillance study consisted of a human health syndromic surveillance study that collected data on fever, diarrhoea, jaundice and respiratory syndromes from all consenting household members [27] and an animal health syndromic surveillance study, started in 2013, that collected routine data on occurrence of nine syndromes from domestic livestock (cattle, sheep, goats and chicken) in the same households participating in the human surveillance. Household socio-economic data were collected from the study households quarterly by a team of trained community interviewers.

### (b) Syndromic data collection

The syndromic data used in these analyses focused on nine animal syndromes (death, reproductive, digestive, musculo-skeletal, skin, urogenital, nervous, udder and respiratory disorders) among the commonly kept livestock species (cattle, goats, sheep and chickens). Livestock ownership in the study households is high (93% of the households own at least one of these livestock). Details are provided in a previous publication [26]. The species under surveillance and main signs defining the nine syndromes are summarized in table 1.

**Table 1.** Description of the nine animal syndromes and the species under investigation in the study.

syndromes	species	characteristics
reproductive	cattle, sheep, goats	abortions, stillbirths, neonatal deaths
gastrointenstinal	cattle, sheep, goats	diarrhoea, constipation, bloating
urogenital	cattle, sheep, goats	haematuria, vaginal/preputial discharges, scrotal swelling
musculo-skeletal	cattle, sheep, goats	lameness, recumbency
skin disorders	cattle, sheep, goats	alopecia, itching, lumps
nervous	cattle, sheep, goats	aggression, incoordination, circling
udder disorders	cattle, sheep, goats	mastitis, decrease in milk production
death	cattle, sheep, goats, chicken/chicks	chicken^a^/chicks^b^

^a^Chicken—mortality classified as death in more than 30% of the chickens over three months old.

^b^Chicks—mortality classified as death in more than 50% of the chickens under three months old.

Surveillance data were collected through two main methods: (i) routine household visits conducted by community interviewers trained on questionnaire administration and electronic data capture using hand-held digital instruments, and (ii) through a mobile phone-based toll-free surveillance system where study participants could call in and report disease events at any time. The cost of calls made by animal owners reporting disease events were incurred by the research study. From February 2013 to May 2015, the routine household visits were conducted bi-weekly and thereafter monthly.

The mobile phone-based surveillance system was set up through purchase of a toll-free line linked to an additional four numbers (referred to as hunting lines), to allow for up to five different callers to call in simultaneously. Each of these lines was managed by a staff member engaged in the surveillance study. Information about the toll-free number was regularly distributed to the study households printed on cards containing a message that instructed the farmers to report illness or death by calling the number displayed on the card at no charge.

Basic mobile phones able to connect by voice only were sufficient; the system did not require smartphones. The average cost of maintaining the toll-free number was USD $200 per month with a provision for up to 40 000 min of toll-free calls.

For each livestock disease event, the source of the report (whether obtained through the routine household visit or through the toll-free number) was recorded. The study veterinary team responded through household visits to all disease event reports within 24 h of receiving the report. During the response visits, the veterinary teams clinically examined the animals to verify the report and provided free treatment for all cases with syndromes under investigation. A case was considered valid if it involved at least one of the nine syndromes in table 1 that had been verified by the veterinary team. Reports received that did not fall under the nine syndromes were considered as invalid. For syndromes falling outside the nine study syndromes such as traumatic injuries, the research team referred the cases to the non-study veterinarians or animal health technicians working in the study area.

Our analysis compared syndromic surveillance data for livestock diseases collected through the household visits and through the mobile phone-based method to investigate the temporal patterns of disease events reported, and accuracy between the two methods. By linking the reported disease events with household socio-economic data collected quarterly, we investigated the determinants of mobile-based surveillance for infectious diseases. Details of the socio-economic data collected have been provided in an earlier manuscript [26].

### (c) Data analysis

Using data from a 5-year period between February 2013 and December 2018, we compared reports of disease from the two surveillance methods by number of reports, their validity and temporal patterns. Using logistic regressions, we tested the likelihood of a valid case (outcome variable as either valid or invalid case) as determined by the reporting method (toll-free number, routine household visit). A similar analytical approach was used to determine the likelihood of reporting animal illness or deaths through each of the two reporting methods. Each event reported was classified either as an illness (Yes or No) or a death event (Yes or No) and these were compared by the surveillance method used to report the event. The analyses were repeated for death and illness events by species, and tested differences in the reporting method used for specific syndromes by livestock species.

We computed the total number of disease events reported by each household using the toll-free number, over the study period. Using multivariable negative binomial linear regressions, we tested whether socio-economic factors, including gender, education level, occupation, age, phone ownership—while controlling for herd and flock sizes—were associated with the likelihood of using toll-free numbers for disease reporting. Each of the putative factors was tested in a univariable analysis and all factors with *p* < 0.2 were included in a multivariable model. Factors found not to significantly increase model fit were dropped and results of the minimum adequate model presented. All data analysis was conducted using R statistical computing environment v. 3.4.2 [28].

## 3. Results

### (a) Characteristics of surveillance data by reporting method

**Figure 1.**
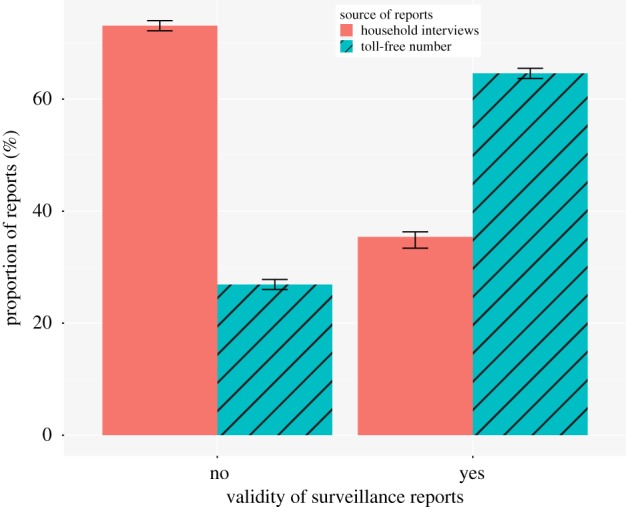
Surveillance reports received through the toll-free number constituted a significantly higher proportion of the valid reports and a significantly lower proportion of invalid reports compared to surveillance reports received through the routine household surveys. (Online version in colour.)

A total of 20 340 reports were received during the study period of which 10 976 (54%) and 9364 (46%) were obtained through the routine household surveys and through calls from farmers using the toll-free number, respectively. Given that these reports were from animal owners as reported to community interviewers (without veterinary training) or directly by animal owners to the veterinary teams through the toll-free number, verification of all reported events was done through household visits by the study veterinary team within 24 h of receiving the report. A total of 10 324 (51%) of all disease events reported were confirmed valid (could be classified into any of the nine study syndromes). Reported events that did not meet the inclusion criteria were considered invalid. Reports received from the household surveys comprised 73% (7322 of 10 016) of all invalid reports, whereas 65% (6670 of 10 324) of the valid disease events were reported through the toll-free phone system. The probability of an event reported through the household survey being valid was 0.33, while that of an event reported through the toll-free phone system was 0.71, indicating the toll-free system was more reliable in identifying valid cases compared to household surveys. Using the logistic regression framework, we coded valid cases as ‘1’ and invalid cases as ‘0’ and tested the odds of valid cases, given the surveillance method used to report the disease. The toll-free phone system was five times (95% CI 4.7, 5.3) more likely to capture valid animal illness and death events compared to the routine household visit surveys collecting animal health data, figure 1.

**Figure 2.**
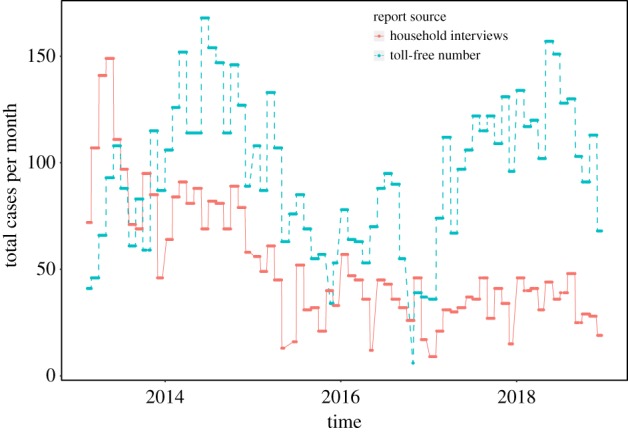
Number of valid animal syndromic surveillance reports by source of report and by month for the period 2013–2018. (Online version in colour.)

In order to compare the number of disease events submitted through the two methods over time, we calculated the monthly cumulative number of reports by each method for the study period 2013–2018 (figure 2). At the start of the surveillance project, most of the data were obtained through the routine household visits, while the use of the toll-free number to report disease events increased as more community members adopted the method. By mid-2013, valid reports of disease events received through the toll-free number surpassed those received through the household visits. The decline in the number of cases reported through both methods in May 2015 resulted from administrative changes that included a shift from bi-weekly to monthly household visits, and a month towards end of 2016 when the toll-free number was inoperative.

### (b) Reporting by type of animal syndromes

We examined the differences in the surveillance data reporting methods by livestock species and type of syndrome. Illness events were 14.8 times (95% CI 12.8, 17.1) more likely to be reported through the mobile phone-based surveillance system when compared with the routine household visits. This was consistent for the three livestock species, cattle, goats and sheep. Conversely, death cases were less likely to be reported through the mobile phone-based surveillance system (OR 0.1, 95% CI 0.09, 0.11) compared to routine household visits (electronic supplementary material, table S1).

**Figure 3.**
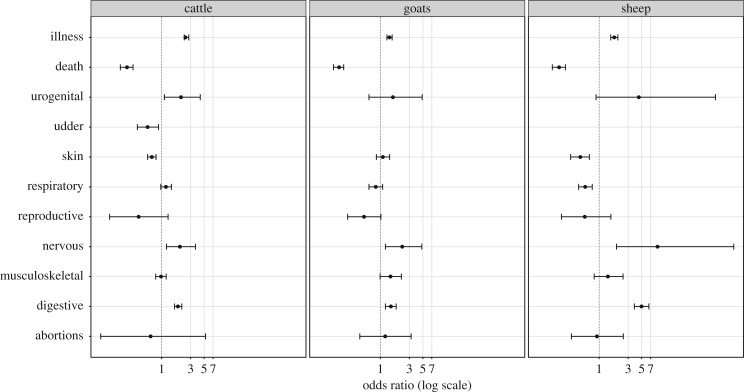
Odd ratios of using the phone-based surveillance system in reporting illness and death events, and different disease syndromes by livestock species compared to reporting through the household visit animal health surveys.

We observed significant differences in the reporting methods according to the type of disease syndrome observed. In the three species (cattle, sheep and goats), the farmers were more likely to report gastrointestinal syndromes such as diarrhoea and nervous syndromes through the mobile phone-based surveillance system compared to reporting through household animal health surveys. Additionally, farmers reported more urogenital cattle syndromes through the mobile phone-based system than through the household visits. Skin conditions were less likely to be reported by mobile phone in cattle and sheep (figure 3). Additional information is provided in electronic supplementary material, tables S1 and S2.

### (c) Household determinants of reporting using the phone-based surveillance system

We investigated the factors associated with the likelihood of farmers using mobile phone reporting for disease syndromes observed on their farms by linking surveillance data received and the data on the socio-economic status of study households. While controlling for the numbers of different livestock species in the study households (larger herd and flock sizes increased the likelihood of disease and death reports), we found households where the household head had not received any formal education, and households with additional sources of income other than farming, had a decreased likelihood of reporting disease events through the phone-based surveillance system. The age of the household head was associated with increased likelihood of phone-based reporting, but neither gender nor phone ownership at the household level were strongly associated with phone-based disease reporting (table 2).

**Table 2.** Results from the multivariable model showing the household-level and individual-level determinants of reporting animal illness and death events through the phone-based surveillance system.

variable	estimate	lower CI	upper CI
household phone ownership (yes)	1.21	0.78	1.88
age of household head (/10 years)	1.08***	1.03	1.12
household head (male)	1.09	0.96	1.23
household head occupation (non-farming income)	0.75***	0.66	0.86
household head education (no formal education)	0.75*	0.59	0.97
cattle herd size	1.27***	1.19	1.35
number of goats	1.12***	1.09	1.15
number of sheep	1.07***	1.04	1.09
poultry flock size	1.01	0.99	1.02

Levels of significance: ‘***’ 0.001; ‘*’ 0.05.

## 4. Discussion

This study demonstrates that the use of a mobile phone-based animal health surveillance system is an effective tool for reporting of disease events by communities in a rural setting in Africa. The study design provided rare longitudinal data allowing for evaluation and comparison of two surveillance systems employed in the same geographical area and households concurrently, used in reporting similar disease events in the same animal species, and with the data corroborated through response visits conducted by trained animal health assistants and veterinarians. Our data show that the phone-based system has a higher probability of reporting valid disease events compared with the household disease surveillance visits, paradoxically demonstrating that a mobile phone-aided passive surveillance system can outperform an active surveillance system. Active surveillance systems such as routine visits to study households require more time and resources than a passive surveillance system where animal owners have to decide whether to report disease events occurring at their farm.

Mobile phones are a routine of most people's everyday life. Their use in supporting public health systems in developing countries to address a lack of quality data and instant transmission of health data from lower levels has been documented [19,29]. Unlike such surveillance systems that depend on healthcare or veterinary workers to report disease events (including those using mobile phones to improve reporting), this study has demonstrated a mobile phone-based surveillance system directly dependent on farmers and community members to report disease events. Our findings that owning a mobile phone is not a determinant of using the phone-based surveillance system are insightful, indicating a good interplay between widespread phone ownership and likelihood of accessing phones to report disease events, even when households do not own phones. This is important as it removes the possible reporting bias that would be associated with mobile phone ownership.

In the past 20 years, Kenya has experienced two major outbreaks of Rift Valley haemorrhagic fever (RVF): the 1997–1998 outbreak that killed 450 people and the 2006–2007 outbreak that killed 158 people [30,31]. In both cases, the disease first appeared in livestock before human cases were reported, but the lower mortality during the 2006–2007 outbreak was attributed to the swift outbreak detection and reporting made possible by mobile phone communication advances, aetiologic confirmation through real-time polymerase chain reaction tests and immediate deployment of public health response [32]. This kind of early detection for Rift Valley Fever and other EID like avian influenza, Nipah encephalitis and severe acute respiratory syndromes (SARS) relies on animal health surveillance to prevent or control spill over to the human population.

Our findings on greater propensity of using the phone-based system for reporting illnesses (especially those presenting with severe clinical signs) and not death events has broader implications for the surveillance for infectious diseases in livestock and how we might implement surveillance for different zoonotic infectious diseases. Diseases that have severe clinical presentations such as the furious form of rabies or haemorrhagic fevers such as Rift Valley fever that may present in animals with multiple signs such as fetid or bloody diarrhoea, listlessness, reluctance to move or feed, abdominal pains, nasal discharges alongside other clinical signs would be sufficiently captured using this system. Conversely, infections such as highly pathogenic avian influenza, which may often result in mortality with few or no prior clinical signs, may be missed by the phone-based surveillance system with observed lower likelihood of using the system for reporting mortality cases.

The effectiveness of surveillance systems is linked to response actions or incentives for reporting. In our study, veterinary responses to all reported cases likely served as an effective incentive for continued community reporting of disease events observed in their animals. Reporting illness cases provided animal owners with opportunities for receiving immediate help for their sick animals without incurring veterinary treatment expenses. Although reporting death cases may lead to knowledge of what killed the animals and what might be done to prevent similar deaths in the future, this was not seen as sufficient incentive for real-time reporting of death events in our study.

Similar challenges on death cases are experienced in public health, especially in developing countries. For example, deaths owing to diseases such as rabies are often unconfirmed as family members may not be interested in authorizing an autopsy even though a definitive diagnosis for the disease can only be reached through post-mortem examination and testing of a brain sample [33]. Data on causes of mortality in humans, even though critical for precision public health, have been difficult to obtain in most developing countries [34].

The phone-based surveillance system in this study was dependent on voice only, and was toll-free, removing the barriers of requiring high-end smartphones and costs associated with data transmission. However, this system requires that there is a person on the other end to receive the phone calls and initiate the veterinary responses. To roll-out phone-based surveillance systems at the community level, a careful balance between adding complexity as use of texting and data collection phone applications, and the simplicity and ease of voice reporting should be considered. Such user experience and usability over technological advances has been recommended as critical in scaling up mobile phone-based health programmes [21].

We argue that community-based surveillance including for zoonotic infectious diseases in animals can be applied across all diseases but requires thought on incentives for infections that would present with subtle or no clinical signs, or with death. This suggests opportunities to identify incentives and socio-cultural determinants of behaviour that play a role in improving surveillance for infectious diseases, and ultimately global health security. The goal of this kind of phone-based surveillance system should be integration within the country animal health surveillance systems, timely analysis of the data collected to detect outbreaks and using a One Health approach including sharing data with human health and other relevant sectors, for appropriate responses that ensure food, economic and health security. Whereas achieving this requires a complex interplay of infrastructural, political, socio-economic and cultural factors, such a system is a critical early step in detecting and preventing zoonoses spillover among livestock-keeping communities in rural areas.

## Supplementary Material

Supplementary tables containing results of odds of reporting animal illness and death events through the mobile phone-based surveillance system

## Data accessibility

The data and code underlying this study are available from Open Science Framework: https://osf.io/8c4xg/. This dataset is available under a CC0 1.0 Universal licence.
