# Supplementary material for: Mobile phone-based surveillance for animal disease in rural communities: implications for detection of zoonoses spillover
Source: Philos Trans R Soc Lond B Biol Sci. 2019 Aug 12;374(1782):20190020. doi: 10.1098/rstb.2019.0020 (PMC6711315; doi:10.1098/rstb.2019.0020)
Supplement: Supplementary tables containing results of odds of reporting animal illness and death events through the mobile phone-based surveillance system [file rstb20190020supp1.docx]

Supplementary Material

**Mobile phone based surveillance for animal disease in rural communities: implications for detection of zoonoses spillover**

Thumbi et al

Supplementary Table 1: Results of univariable analysis showing odd ratios of reporting illness and death through the toll-free number compared to through routine household surveys by livestock species.

|  | Estimate | Lower CI | Upper CI |
| --- | --- | --- | --- |
| Reports of illness |  |  |  |
| Toll-free number (all species) | 14.78 | 12.82 | 17.1 |
| By species (toll-free) |  |  |  |
| Cattle | 2.57 | 2.35 | 2.8 |
| Goats | 1.41 | 1.28 | 1.56 |
| Sheep | 1.77 | 1.55 | 2.03 |
| Reports of death |  |  |  |
| Toll-free number (all species) | 0.1 | 0.09 | 0.11 |
| By species (toll-free) |  |  |  |
| Cattle | 0.27 | 0.21 | 0.34 |
| Goats | 0.21 | 0.17 | 0.25 |
| Sheep | 0.22 | 0.17 | 0.28 |
| Chicken | 0.06 | 0.05 | 0.09 |
| Chicks | 0.02 | 0.01 | 0.03 |

Supplementary Table 2: Results of univariable analysis showing odd ratios of reporting different disease syndromes among livestock species using the mobile phone-based surveillance system compared to routine household visits.

|  | Estimate | Lower CI | Upper CI |
| --- | --- | --- | --- |
| Cattle syndromes |  |  |  |
| Reproductive | 0.42 | 0.14 | 1.28 |
| Respiratory | 1.18 | 0.97 | 1.45 |
| Digestive | 1.86 | 1.63 | 2.15 |
| Urogenital | 2.08 | 1.11 | 4.32 |
| Musculoskeletal | 0.98 | 0.8 | 1.19 |
| Nervous | 2 | 1.2 | 3.6 |
| Udder | 0.59 | 0.4 | 0.89 |
| Skin | 0.69 | 0.59 | 0.81 |
| Abortions | 0.66 | 0.1 | 5.27 |
| Goat syndromes |  |  |  |
| Reproductive | 0.54 | 0.29 | 1.02 |
| Respiratory | 0.84 | 0.65 | 1.09 |
| Digestive | 1.49 | 1.21 | 1.82 |
| Urogenital | 1.61 | 0.65 | 4.86 |
| Musculoskeletal | 1.47 | 0.99 | 2.21 |
| Nervous | 2.29 | 1.21 | 4.79 |
| Skin | 1.1 | 0.86 | 1.42 |
| Abortions | 1.2 | 0.46 | 3.2 |
| Sheep syndromes |  |  |  |
| Reproductive | 0.58 | 0.24 | 1.56 |
| Respiratory | 0.59 | 0.46 | 0.77 |
| Digestive | 4.98 | 3.79 | 6.57 |
| Urogenital | 4.49 | 0.89 | 81.45 |
| Musculoskeletal | 1.39 | 0.83 | 2.47 |
| Nervous | 9.1 | 1.93 | 162.7 |
| Skin | 0.49 | 0.34 | 0.69 |
| Abortions | 0.92 | 0.35 | 2.49 |
